# Results of a Pilot Study on the Safety and Early Efficacy of Novel Polymethyl Methacrylate Microspheres and a Hyaluronic Acid Device for the Treatment of Low Back Pain Caused by Degenerative and Diseased Intervertebral Discs of the Lumbar Spine

**DOI:** 10.7759/cureus.16308

**Published:** 2021-07-11

**Authors:** James Yu, William Taylor, Paul Verrills, Lachlan R Porter, Alex Gavino, Jacqui F Young, Masego Johnstone, Donald Ponec, Niv Caviar, Mihir Mehta

**Affiliations:** 1 Pain Medicine, Australian Medical Research, Sydney, AUS; 2 Neurosurgery, University of California San Diego School of Medicine, Del Mar, USA; 3 Pain Medicine, Metro Pain Group, Melbourne, AUS; 4 Clinical Research, Mobius Medical, Sydney, AUS; 5 Pain Medicine, Monash Clinical Research Pty Ltd, Melbourne, AUS; 6 Interventional Radiology, San Diego Imaging, Carlsbad, USA; 7 Administration, SpineOvations, Carlsbad, USA; 8 Biomedical Engineering, SpineOvations, Linden, USA

**Keywords:** chronic low back pain (clbp), clinical trial, first in human, discogenic back pain, degenerative disc disease

## Abstract

Background

Low back pain (LBP) costs the healthcare system billions of dollars each year. Intervertebral disc (IVD) degeneration is a significant cause of LBP, due to structural defects, biomechanical instability, and inflammation. First-line therapy for patients with LBP includes physical therapy, medication, and steroid injections. DiscSeal^TM^ was developed to provide patients who are refractory to first-line therapy with a minimally invasive treatment alternative to invasive surgical procedures. The product is a combination of poly-methyl methacrylate (PMMA) microspheres in hyaluronic acid (HA) that is injected under modified discography into the IVD.

Methods

Two pain specialist centers in Australia recruited eligible participants who were followed up for 180 days post-procedure. The procedure was conducted using a modified discography technique. Low back and leg pain was reported using the Visual Analogue Scale (VAS) while other endpoints included were Oswestry Disability Index (ODI), Clinician and Patient Global Impact of Change, and Patient Rating of Overall Health Status. The general analytical approach for all endpoints was descriptive in nature and 95% confidence intervals of means were estimated.

Results

The pilot study achieved its primary objective which was an absence of peri-treatment or post-treatment device-related Serious Adverse Events (SAE) during the first 90 days. There were no device-related serious adverse events recorded throughout the study. The mean LBP percentage change from baseline at 90 and 180 days was -27.0% and -42.3% respectively. The mean ODI percentage change from baseline at 90 and 180 days was -22.3 and -14.2% respectively. End of study improvements shows a 67.8% (20.83) increase in Overall Health Status, as well as positive results for Participant and Clinician Global Impact of Change. These results were achieved based on treating one diseased IVD, although 83.3% of patients were diagnosed with multiple diseased IVDs.

Conclusions

The results from this pilot study showed that DiscSeal^TM ^is safe and well-tolerated. Early efficacy shows that DiscSeal^TM ^may be a promising treatment option for people suffering from discogenic LBP that have not responded to first-line treatment options. A larger, statistically powered study where all diseased discs are treated should be completed to validate the promising results from this early feasibility study.

## Introduction

Low back pain (LBP) is a major healthcare problem causing more disability than any other medical condition [1]. In developed countries, LBP is the second most common reason where patients seek medical treatment after a cough [2]. Patients with LBP experience pain, disability and loss of productivity leading to significant psychological, social, and economic burden. The direct and indirect costs of LBP in the United States alone are greater than $100 billion per year [1]. The lifetime prevalence of LBP is reported to be 60 - 70% [3]. LBP is a World Health Organisation priority disease, with incidence and burden on health resources expected to increase as the world population ages [3].

Intervertebral disc (IVD) disease is a significant cause of pain in LBP patients and involves degenerative changes to the IVD leading to structural defects, biomechanical instability, and inflammation [4]. 

First-line therapy for patients with LBP includes physical therapy, oral pain relievers, and steroid injections [5]. Treatment options for patients who fail first-line therapy are limited to invasive surgical procedures such as total disc replacement or spinal fusion [6]. The recovery from such surgical procedures can be lengthy and may result in a permanent loss of flexibility. Many surgical approaches for LBP have limited or transient efficacy and there is little evidence to show which procedures work best for particular indications [4]. Hence, minimally invasive treatment options for LBP are urgently needed. 

This study aimed to investigate the safety, tolerability, and exploratory efficacy of a novel intra-discal formulation for the treatment of degenerative and diseased intervertebral disc (IVD) of the lumbar spine. The primary objective of the study was to show freedom from any peri-treatment or post-treatment device-related Serious Adverse Events (SAEs) during the first 90 days. A secondary aim of the study was to test the efficacy of the DiscSeal^TM^ device.

## Materials and methods

Investigational product

The DiscSeal^TM^ product platform is based on repurposing of dermal fillers and is a colloidal form of a viscous gel composed of cross-linked Poly (methyl methacrylate) (PMMA) microspheres (with diameters between 53-106 μm) and a proprietary formulation of aqueous buffered, high molecular weight sodium hyaluronate (HA), supplied as sterile 1.0 mL, single-use, pre-filled syringes. The intended mode of action of the product is to be injected directly into the nucleus pulposus (NP) of the IVD of the lumbar spine with the PMMA microspheres providing structural support, volume, bulk, and will potentially seal annular fissures of the IVD. DiscSeal^TM^ could potentially also retard or delay further degradation or continued loss of IVD height and/or volume. The HA in DiscSeal^TM^ is biodegradable and is intended to serve as a carrier for the microspheres, provide transition filling and flow properties, and supply viscosity for injection and retention of PMMA in the NP. The HA selected for DiscSealT^M^ is not cross-linked and is made from recombinant processes and does not contain animal-derived products therefore there is no risk of transmissible spongiform encephalopathies (TSE)/bovine spongiform encephalopathy (BSE). It is believed the HA will be biologically absorbed within a few weeks of the procedure, leaving the PMMA microspheres within the NP. Phosphate Buffered Saline (PBS) and water are added as the aqueous buffer. It is postulated that the microspheres remaining in the IVD are either encapsulated by fibrotic tissue or native new collagen.

The biocompatibility and biological safety of DiscSeal^TM^ has been investigated in several animal models. Large animal studies involving sheep resulted in no adverse health or neurological findings when followed up for eight months. Necropsy observations revealed no visible lesions or other abnormalities. Based on the histopathologic analysis, it was concluded that DiscSeal^TM^ appears to be safe at eight months post-treatment via IVD injection with no evidence of tissue reaction/inflammation at the treated IVD. DiscSeal^TM^ also appeared to stay localized primarily in the NP and annulus fibrosus (AF) of treated IVDs. DiscSeal^TM^ also appeared to have no adverse effects on adjacent tissues and there was no migration of microspheres in any of the animal studies performed.

PMMA and HA are components of a large number of devices that have regulatory clearance by the Food and Drug Administration, European Medicines Agency, and Therapeutic Goods Australia and their safety profile is well known. Percutaneous vertebroplasty and kyphoplasty vertebral augmentation procedures with PMMA based bone cement have been widely used to treat painful vertebral compression fractures for decades with leaks outside the confines of the vertebral body often occurring but rarely resulting in clinical sequelae. PMMA in DiscSeal^TM^ is delivered in much smaller volumes and as preformed, hardened microspheres, designed to decrease the risk of leakage, instead of widely used liquid, non-cured PMMA.

Study design

The study was a prospective, single-arm feasibility clinical study conducted at two Australian centers between 30 September 2019 and 22 January 2020. The study was reviewed and granted approval by Bellberry Ltd Human Research Ethics Committee (HREC Application No. 2019-07-595) prior to recruitment, and was prospectively registered in the WHO Primary Registry and Australia New Zealand Clinical Trials Registry (ANZCTR) (Trial ID: ACTRN1269001145190).

Participant selection

Males and females aged between 18 to 70 years were recruited if they had been diagnosed with chronic LBP (VAS ≥ 4) for at least six months where the etiology of their pain was predominantly discogenic in the lumbar spine (Degenerative Disc Disease, Internal Disc Disruption, or nonspecific Discogenic Disease) and they had failed at least six weeks of conservative management. Refer to Table 1 for the full list of eligibility criteria.

**Table 1:** Eligibility Criteria for the DiscSeal Investigation

Inclusion Criteria	
1	Male or female between the age group of 18- 70 years (inclusive); If female, must have a negative pregnancy test at the time of treatment, be actively practicing contraception or abstinence, be surgically sterilized, or be postmenopausal.
2	Participant presently has degenerative disc disease, as evidenced by
	I. History and clinical findings suggestive of degenerative disc disease (DDD), internal disc disruption (IDD) or non-specific discogenic disease
	II. At least Modic Type 1 changes on MRI [7]
	III. Disc height of ≥50% of normal disc height (defined as the average height of adjacent normal discs) based on anteroposterior and lateral lumbar spine radiographs (plain X-ray images)
3	Visual Analog Scale (VAS) low back pain score of back pain of at least ≥ 4 [8]
4	Oswestry Disability Index (ODI) >21% (i.e., at least moderate disability) [9]
5	At least one lumbar disc with Pfirrmann Grade II-IV or Modic Type 1-2 changes without annular rupture [7]
6	No annular tears present in the disc planned for treatment which reach the distal periphery of the annulus with the potential for being incapable of holding the DiscSeal
7	Tried and failed at least six weeks of conservative management as directed by a licensed physician, chiropractor, and/or physical therapist. Treatment must include any or a combination of physical therapy, chiropractic care, or pain management, including but not limited to, rest or activating physical therapy, heat, cold, electrical stimulation, ultrasound, manipulation, acupuncture, analgesics including narcotics (with no history of abuse), anti-inflammatory medication, radiofrequency treatments, and spinal injections, including epidural steroid, facet joint injection and or anesthesia injections
8	Participants who are legally competent and able to understand the nature, scope and aim of DiscSeal
9	Participant is on a stable dose (no new, discontinued, or changes in dose) of all prescribed pain medication for at least 30 days prior to baseline evaluation
10	Participant is willing to remain on the current medication regimen for the next 90 days following the investigational procedure
11	Participant has experienced Chronic low back pain for at least six months.
Exclusion Criteria	
1	Body mass index (BMI) ≥40 kg/m2 at the Screening Visit.
2	Disc height of <50% of normal disc height (defined as average height of adjacent normal discs) based on anteroposterior and lateral lumbar spine radiographs (plain X-ray images)
3	More than one Pfirrmann Grade III or IV lumbar disc [10]
4	Pfirrmann Grade V lumbar disc at any level in the lumbar spine [10]
5	Current disc extrusion at any level in the lumbar spine unsuitable for treatment in the opinion of the investigator as judged during discography
6	Disc bulges or protrusions at any level in the lumbar spine resulting in radiculopathy
7	Osteoporotic compression fracture at any vertebral level
8	Lumbar Scheurmann disease or other significant wedge deformity or malalignment at the targeted level
9	Anterolisthesis or retrolisthesis ≥ 3 mm at any level
10	Moderate to severe facet disease at any level of the lumbar spine, at the investigator’s discretion
11	Symptomatic central canal stenosis or symptomatic foraminal stenosis
12	Spondylolysis or instability at the level targeted for treatment
13	Lumbar coronal angulation ≥ 10°
14	Cauda equina syndrome
15	Extradiscal extravasation of contrast on discogram
16	Participants who cannot tolerate modified discography
17	Previous spine surgery or other invasive treatment of the study disc, with the exception of previous epidural steroid, platelet-rich plasma (PRP) injection (not within six months), or anesthesia injection
18	Currently experiencing chronic pain generating from any other source that (in the judgment of the investigator) may interfere with the evaluation of back pain, and or back pain-related disability and/or physical well being
19	Radicular pain >50% of low back pain (as evidenced by nerve root tension signs) and/or radiculopathy or participants that have primary radicular pain due to nerve compression
20	Scoliosis
21	Active systemic infection or infection at the operative site, as evidenced by a brief physical examination
22	Suffers from rheumatoid arthritis or other autoimmune disease or a systemic disorder such as HIV, active hepatitis B or C, or fibromyalgia
23	Has a medical condition (e.g., unstable cardiac disease, cancer) that may result in participant death or have an effect on outcomes prior to study completion
24	Has a physical or mental condition (e.g., psychiatric disorder, senile dementia, Alzheimer's disease, alcohol or drug addiction) that would interfere with participant self-assessment of function, pain, or quality of life.
25	Incarcerated at the time of study enrolment
26	Has been enrolled in a clinical investigation for the treatment of intervertebral disc disease or received a study drug or investigational biological agent for the treatment of intervertebral disc disease within the last 180 Days
27	Has been enrolled in any other clinical investigation in the past 90 days, is currently enrolled in any other clinical investigation, or is planning to be enrolled in any other clinical investigation during the complete study period
28	Any known allergy to the contrast agent
29	Pregnancy
30	Opioid use greater than an average of 30 mg morphine equivalents per day in the two weeks prior to the Baseline Visit.
31	Unwilling or incapable of discontinuing any of the medications listed in the prohibited medication list
32	Any known allergy to sodium hyaluronate or polymethyl methacrylate (PMMA)
33	Diagnosed with 3 or more severely diseased discs requiring treatment
34	Planned surgical lumbar disc surgery required within the duration of this clinical study

Study procedure

All study participants were consented prior to screening. Medical history and concomitant medications were recorded. Baseline assessments included a full physical examination, vital signs, and the following screening questionnaires: Oswestry Disability Index (ODI), Visual Analog Scale (VAS) for pain, and Overall Health Score (OHS). Magnetic resonance imaging (MRI), and X-rays of the lumbar spine that were no less than six months old were used to identify disc degeneration. Participant data were reviewed by an independent medical monitor to assess eligibility. All eligible participants were enrolled for treatment.

DiscSeal^TM^ injections were performed by qualified and experienced investigator clinicians who used their own standard of care to prepare and manage the participant for the procedure (including infection and pain control). Procedures were conducted under general sedation at the discretion of the investigator. Treatment was administered using ‘modified discography’, where a minimum amount of contrast was first injected into the IVD to determine if the disc could contain DiscSeal^TM^ in situ and to confirm needle tip placement for the optimal injection process. As an additional safeguard, the DiscSeal^TM^ material, which is not radiopaque, was transferred into a sterile receiving syringe prior to injection to ensure adequate flow and viscosity characteristics of the product. DiscSeal^TM^ was injected into the target disc’s nucleus under fluoroscopic image guidance via a 20 g Chiba needle at a rate similar to that of contrast injection during discography. DiscSeal^TM^ was delivered until the investigator felt that the disc could not hold any more or the full 2.0 mL was administered. A target amount of 1.0 to 2.0 mL was described as it is the common range for other intradiscal treatments and the goldilocks amount that would have an effect without going beyond the tactile injection pressures.

A two-week pause was observed prior to doing a subsequent procedure for the first two participants to allow the independent Medical Monitor to perform a safety review.

Participants treated with DiscSeal^TM^ were followed for 180 days, with study visits at seven days, 14 days (phone check-up for safety), 42 days, 90 days, and 180 days post-treatment (refer to Table 2 for the full study schedule). The 90-day visit was the primary endpoint of the study, with the focus on confirming that participants had suffered no serious adverse effects from the investigational device or procedure. An additional 180-day visit was included to demonstrate the longer-term safety of the DiscSeal^TM^ implant. Participant follow-up visits included a full physical examination, vital signs, concomitant medications, and a check for adverse events. Assessments of device effectiveness included low back and leg VAS, ODI, OHS, Clinical Global Impression of Change (CGI-c) questionnaire, and the Patient Global Impression of Change (PGI-c) questionnaire. A follow-up MRI was performed for all participants at either 90 days or 180 days post-index procedure as an additional safety measure.

**Table 2:** Study Schedule Indicating the Assessments Performed at Each Visit a. Final eligibility will be confirmed through modified discography, b. The pre-operative imaging data (MRI and X-rays) can be obtained up to 180 Days prior to Treatment, c. Administered pre-treatment. May be collected over the telephone where necessary, d. All willing participants will have an MRI performed at either their 90-day follow-up visit or 180-day follow-up visit, e. Any additional assessments collected at unscheduled visits will be included in case report forms, f. Contact by phone. The patient is assessed for resumption to pre-procedure activity level, adverse events, and medications. If the patient requires an office visit, this will be classed as an unscheduled visit. VAS- Visual Analogue Scale, ODI- Oswestry Disability Index, CGI-c - Clinical Global Impression of Change, PGI-c - Patient Global Impression of Change.

Schedule of events	Visit 1	Visit 2	Visit 3	Visit 4	Visit 5	Visit 6	Visit 7	Unscheduled Visits ^e^
	Screening	Treatment Day 1	Follow-up Day 7	Follow-up Day 14	Follow-up Day 42	Follow-up Day 90	End of Study Day 180	
	≤ 90 days		-2 to +2 days	-2 to +2 days	± 7 days	± 14 days	± 30 days	
Site Visit	X	X	X	optional^ f^	X	X	X	X
Phone visit				X^ f^				
Informed Consent	X							
Eligibility Criteria	X	X^a^						
Demographics/ Medical History	X							
Brief Physical examination	X		X		X	X	X	X
Assess vital signs	X	X	X		X	X		X
Concomitant medications	X	X	X	X	X	X	X	X
Modified Discogram		X						
MRI	X^b^					X^d^	X^d^	
Radiographs (Plain X-Ray)	X^b^							
VAS score for Back/leg pain	X	X^c^	X		X	X	X	
ODI questionnaire	X	X^c^	X		X	X	X	
CGI-c questionnaire			X		X	X	X	
PGI-c questionnaire			X		X	X	X	
Patient Rating of Overall Health Status		X^c^	X		X	X	X	
Device implantation		X						
Assessment of activities of daily living				X				X
Adverse Events		X	X	X	X	X	X	X

Statistical analysis

While the study was not powered for sample size calculations, the number of participants enrolled in the study was considered sufficient to adequately characterize the general safety profile of this type of device. Hence, descriptive statistics were used to assess mean, range, median, minimum and maximum for continuous variables and frequency counts and percentages for categorical variables. Missing values were not imputed, and baseline values were defined as the last available non-missing assessment prior to DiscSeal^TM^ injection.

Secondary endpoints were summarized by study visit for the total score, absolute change from baseline, and percentage change from baseline with 95% confidence intervals of the mean estimated. 

Three patient populations were defined pre-study for investigational analysis post-treatment. The Safety Population was to include all participants who received the DiscSeal^TM^ injection. The Intent to Treat (ITT) population was to include all participants who received the DiscSeal^TM^ injection and had at least one post-baseline measurement of VAS or ODI. The Per Protocol (PP) Population was to be a subset of the ITT population without major protocol deviations likely to affect the outcome.

## Results

Ten participants consented to the study, of which four did not meet the eligibility criteria. Six participants were enrolled for treatment with DiscSeal^TM^. The mean participant population age was 38.8 ± 10.0 years, and the majority of subjects were male (83.3%, 5/6), and Caucasian in ethnicity (83.3%, 5/6). The mean height, weight, and BMI of participants were 174.3 ± 7.4 cm, 86.5 ± 17.7 kg, and 28.2 ± 4.2 kg/m^2^ respectively. Full demographic information is available in Table 1. Five out of six (83%) participants were assessed with having multi-disc disease. However, each participant had only one disc treated with DiscSeal^TM^ at the request of the ethics committee. The L4/L5 lumbar disc was treated in four participants, while two participants had their L5/S1 discs treated. The mean procedure time was 10.8 minutes with a range between 6-27 minutes. Refer to Table 3 for details around the procedure in the clinical investigation. 

**Table 3:** DiscSeal First-in-Human Procedural Information SD: Standard Deviation, n: Number of participants

		All Participants (N=6)
Number of Participants		6
Number of Discs Treated		6
Level Treated	L4/L5	4 (66.7%)
	L5/S1	2 (33.3%)
Delivered DiscSeal^TM^ Volume (mL)	n	6
	Mean	1.6
	SD	0.4
	Median	1.6
	Minimum	1.1
	Maximum	2.0
Contrast Delivered (mL)	n	6
	Mean	0.4
	SD	0.2
	Median	0.3
	Minimum	0.2
	Maximum	0.7
Procedure Time (min)	n	6
	Mean	10.8
	SD	8.0
	Median	8.0
	Minimum	6
	Maximum	27

All participants who underwent the DiscSeal^TM^ procedure were included in the Safety Population. The ITT population included all subjects in the study, and the PP population included all except one. The participant excluded from the PP population (Participant 02-002) exceeded the daily morphine equivalent dosage of oxycodone for an operation post-procedurally which met exclusion criteria 30 (Table 1). Data will be reported with a distinction between ITT and PP populations.

The primary objective of the study was achieved and there were no peri- or post-treatment device-related SAEs throughout the study. Adverse events were recorded from the time of participant consent until study exit. A total of 16 adverse events were reported across all study participants (refer to Table 4 which lists all Adverse Events reported for the study). Serious Treatment-Emergent Adverse Events (TEAE) were also captured throughout the trial. A total of two serious TEAE were experienced in 2 of 6 (33.3%) participants through the end of the study. Participant 01-006 presented with Caecal Volvulus which required surgical intervention. Participant 02-002 underwent the protocol required follow-up MRI which revealed a TEAE intervertebral disc protrusion at postoperative day 160 and the election to perform a microdiscectomy by a neurosurgeon. Both TEAEs were categorized as serious based on the ISO 14155 definition. Upon final adjudication by the independent medical monitor, neither serious TEAE was determined to be related to the study device or the procedure. No patients reported any lasting clinical effects or symptoms related to their AEs or SAEs post medical reporting and treatment.

**Table 4:** Treatment-Emergent Adverse Events Reported TEAE: Treatment-Emergent Adverse Events, MedDRA Version: 22.1

	All Participants (N=6)
System Organ Class (SOC) / Preferred Term (PT)	Subjects (%)/Number of AEs
Subjects with at least one TEAE	6(100.0%)/16
Musculoskeletal and connective tissue disorders	5(83.3%)/9
Back pain	4(66.7%)/4
Arthralgia	2(33.3%)/2
Intervertebral disc protrusion	1(16.7%)/1
Musculoskeletal pain	1(16.7%)/1
Neck pain	1(16.7%)/1
Gastrointestinal disorders	3(50.0%)/3
Constipation	1(16.7%)/1
Volvulus	1(16.7%)/1
Vomiting	1(16.7%)/1
General disorders and administration site conditions	1(16.7%)/1
Pain	1(16.7%)/1
Infections and infestations	1(16.7%)/1
Nasopharyngitis	1(16.7%)/1
Skin and subcutaneous tissue disorders	1(16.7%)/1
Blister	1(16.7%)/1
Vascular disorders	1(16.7%)/1
Hypotension	1(16.7%)/1

**Figure 1:**
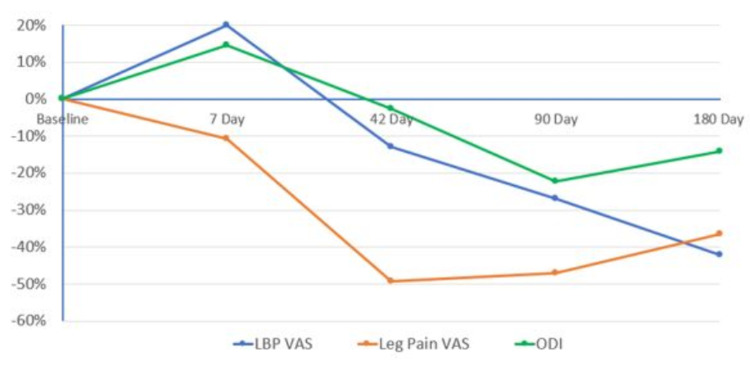
Visual Analogue Score for Low back pain and Leg pain and Oswestry Disability Index Score for the Intent to Treat Population (all Participants) LBP - Low back pain, VAS - Visual Analogue Scale, ODI - Oswestry Disability Index

Improvements in VAS scores, ODI, PGI-c, CGI-c, and OHS compared to baseline were assessed for the secondary objective of the study. For the ITT Population, the mean ODI percentage change from baseline was -22.3% and -14.2% at 90 and 180 days respectively (refer to Table 5 and Figure 1).

**Table 5:** Intent to Treat and Per Protocol Populations' Summary of Oswestry Disability Index (ODI) and Improvements from Baseline SD: Standard Deviation, N: Number of Participants

Visit	Intent to Treat Population			Per Protocol Population		
	Statistics (N=6)	Absolute Value	Percent Change from Baseline (%)	Statistics (N=5)	Absolute Value	Percent Change from Baseline (%)
Baseline	Mean	36.7	--	Mean	38.4	--
	SD	11.0	--	SD	11.4	--
Day 7	Mean	42.0	14.7	Mean	42.4	9.0
	SD	14.1	19.8	SD	15.7	15.9
Day 42	Mean	34.7	-2.5	Mean	34.0	-10.2
	SD	17.7	49.7	SD	19.7	51.4
Day 90	Mean	28.7	-22.3	Mean	25.2	-39.6
	SD	20.6	54.6	SD	21.0	38.4
Day 180	Mean	30.0	-14.2	Mean	27.6	-27.0
	SD	19.9	56.5	SD	21.28	52.5

**Figure 2:**
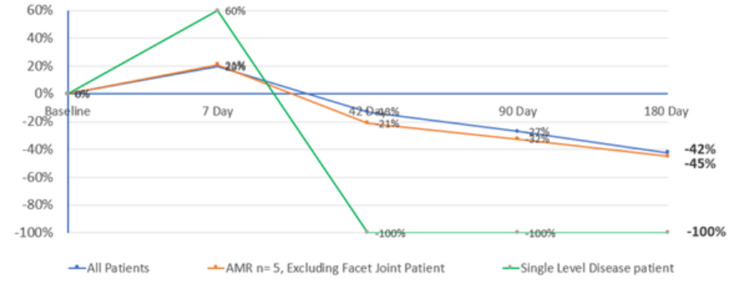
Visual Analogue Scores for Low Back Pain Over Time for all Patients, the Per Protocol Population and Single Disc Diseased Participant

The mean LBP VAS percentage change from baseline was -27.0% and -42.3% at 90 and 180 days respectively (refer to Table 6 and Figure 2). The mean LBP VAS decreased every subsequent visit made to the clinical site after the first visit. The mean leg pain VAS percentage change from baseline was -47.0% and -36.6% at 90 and 180 days respectively (refer to Table 7). End of study improvements in OHS increased 67.8% (Table 8), as well as positive results for Patient and Clinician Global Impact of Change, which are represented in Table 9 and Table 10, respectively. In the PP Population, improvements from baseline were generally greater. Mean ODI percentage change from baseline was -39.6% and -27% at 90 and 180 days respectively (Table 5). The overall mean LBP VAS percentage change from baseline was -32.4% and -45% at 90 and 180 days respectively (Table 6), and the percentage change from baseline of OHS was 86.3% (Table 8). The overall mean leg pain VAS percentage change from baseline was -56.4% and -38.14% at 90 and 180 days respectively (Refer to Table 7). Physical examination and vital sign changes were unremarkable.

**Table 6:** Intent to Treat and Per Protocol Populations' Summary of Low Back Pain Visual Analogue Scale (VAS) and Improvements from Baseline SD - Standard Deviation, N-Number of Participants

Visit	Intent to Treat Population			Per Protocol Population		
	Statistics (N=6)	Absolute Value	Percent Change from Baseline (%)	Statistics (N=5)	Absolute Value	Percent Change from Baseline (%)
Baseline	Mean	5.5	--	Mean	5.2	--
	SD	1.2	--	SD	1.1	--
Day 7	Mean	6.5	19.9	Mean	6.2	21.0
	SD	1.6	28.5	SD	1.64	31.7
Day 42	Mean	5.0	-12.7	Mean	4.2	-21.1
	SD	3.2	45.7	SD	2.9	45.7
Day 90	Mean	4.0	-27.0	Mean	3.4	-32.4
	SD	2.7	48.0	SD	2.5	51.6
Day 180	Mean	3.3	-42.3	Mean	3.0	-45.0
	SD	2.6	38.8	SD	2.7	42.7

**Table 7:** Intent to Treat and Per Protocol Populations' Summary of Leg Pain Visual Analogue Scale (VAS) and Improvements from Baseline SD - Standard Deviation, N-Number of Participants

Visit	Intent to Treat Population			Per Protocol Population		
	Statistics (N=6)	Absolute Value	Percent Change from Baseline (%)	Statistics (N=5)	Absolute Value	Percent Change from Baseline (%)
Baseline	Mean	4.8	--	Mean	4.4	--
	SD	1.9	--	SD	1.8	--
Day 7	Mean	4.2	-6.6	Mean	3.4	-10.7
	SD	2.6	52.7	SD	2.07	57.8
Day 42	Mean	3.2	-49.4	Mean	2.0	-65.0
	SD	4.0	58.0	SD	3.1	48.7
Day 90	Mean	2.8	-47.0	Mean	2.0	-56.4
	SD	2.6	46.1	SD	1.87	44.6
Day 180	Mean	3.5	-36.6	Mean	3.2	-38.14
	SD	2.9	44.8	SD	3.1	49.9

**Table 8:** Intent to Treat and Per Protocol Populations' Summary of Overall Health Status (OHS) and Improvement from Baseline SD - Standard Deviation, N-Number of Participants

Visit	Intent to Treat Population			Per Protocol Population		
	Statistics (N=6)	Absolute Value	Percent Change from Baseline (%)	Statistics (N=5)	Absolute Value	Percent Change from Baseline (%)
Baseline	Mean	47.8	--	Mean	41.4	--
	SD	19.8	--	SD	13.3	--
Day 7	Mean	40.8	-6.9	Mean	40.0	0.5
	SD	8.6	24.3	SD	9.4	18.3
Day 42	Mean	55.3	36.2	Mean	60.0	55.4
	SD	23.0	86.0	SD	22.4	80.4
Day 90	Mean	61.3	55.2	Mean	67.2	78.2
	SD	26.9	105.8	SD	25.4	100.0
Day 180	Mean	68.7	67.8	Mean	70.4	86.3
	SD	17.0	87.5	SD	18.4	83.7

**Table 9:** Intent to Treat and Per Protocol Populations' Summary of Patient Global Impression of Change (PGI-c) SD - Standard Deviation, N-Number of Participants

Visit	Intent to Treat Population		Per Protocol Population	
	Statistics (N=6)	Absolute Value	Statistics (N=5)	Absolute Value
Baseline	Mean	--	Mean	--
	SD	--	SD	--
	PGI-c Improvement (<=3)	--	PGI-c Improvement (<=3)	--
Day 7	Mean	4.8	Mean	4.8
	SD	0.4	SD	0.4
	PGI-c Improvement (<=3)	0	PGI-c Improvement (<=3)	0
Day 42	Mean	4.0	Mean	3.4
	SD	2.2	SD	1.82
	PGI-c Improvement (<=3)	3	PGI-c Improvement (<=3)	3
Day 90	Mean	2.7	Mean	2.2
	SD	1.5	SD	1.1
	PGI-c Improvement (<=3)	5	PGI-c Improvement (<=3)	5
Day 180	Mean	3.3	Mean	2.8
	SD	1.9	SD	1.5
	PGI-c Improvement (<=3)	4	PGI-c Improvement (<=3)	4

**Table 10:** Intent to Treat and Per Protocol Populations' Summary of Clinical Global Impression of Change (CGI-c) SD - Standard Deviation

Visit	Intent to Treat Population		Per Protocol Population	
	Statistics (N=6)	Absolute Value	Statistics (N=5)	Absolute Value
Baseline	Mean	--	Mean	--
	SD	--	SD	--
	CGI-c Improvement (>=1)	--	CGI-c Improvement (>=1)	--
Day 7	Mean	0.3	Mean	0.6
	SD	0.8	SD	0.6
	CGI-c Improvement (>=1)	3	CGI-c Improvement (>=1)	3
Day 42	Mean	0.5	Mean	1.0
	SD	1.8	SD	1.41
	CGI-c Improvement (>=1)	4	CGI-c Improvement (>=1)	4
Day 90	Mean	1.5	Mean	2
	SD	1.5	SD	1
	CGI-c Improvement (>=1)	5	CGI-c Improvement (>=1)	5
Day 180	Mean	0.8	Mean	1.2
	SD	1.5	SD	1.3
	CGI-c Improvement (>=1)	3	CGI-c Improvement (>=1)	3

## Discussion

This first-in-human, interventional, pilot study shows that the novel formulation of PMMA microspheres and HA (DiscSeal^TM^) is a safe medical device that may be used for the treatment of discogenic LBP. To determine safety, the study’s primary objective, participants were closely monitored for adverse events. The 16 adverse events (AEs) that were reported were comparable to the AEs noted in other, similar intradiscal interventions [11] and were expected considering the close scrutiny under which participants were placed. Both SAEs reported were determined to be unrelated to the study device or procedure. AEs that were reported as being related to the study procedure and/or device were expected (for example, localized pain at the injection site). The results from the DiscSeal^TM^ safety study are comparable with other injectables such as platelet-rich plasma (PRP) with a sustained reduction in LBP (42.3% decrease) at six months post-treatment [11-14], and a significant improvement for patients who are refractory to first-line therapy but have no alternative other than to continue on oral, Rx or pain management with limited success, -10.6 out of 100 points VAS reduction with conservative care management [14]. 

Participant satisfaction was also in line with other studies with 33.3% of participants reporting minimal improvement, while 16.7% reported their pain much improved and another 16.7% reported that their pain very much improved at the end of the study. These results are promising considering the confounding complications that occurred with some participant data. 

Participant 01-006 had her results complicated due to confounding circumstances. The participant had to flee from a bushfire prior to the 42-day follow-up and reported an adverse event of increased low back pain from lifting heavy objects whilst trying to save their house. The participant reported an 8 out of 10 low back pain during the 42-day visit which reduced markedly to 3 out of 10 at the 90-day visit. The participant returned to work as a police officer after the 90-day visit and subsequently reported a higher LBP VAS of 7 out of 10 at the 180-day visit.

Mixed results were also seen for the participant 01-004 who reported a 4 out of 10 for LBP at day 42 followed by a 7 at day 90 and a 1 out of 10 at day 180. The participant did report an AE prior to the 90-day visit for increased pain on the hip and low back due to overexerting themselves at the gym.

Participant 02-002 was removed from the per-protocol population, primarily due to taking too much of the prohibited medication oxycodone. The medication was given for pain relief after a microdiscectomy procedure carried out for the SAE “L4/L5 disc extrusion”. 02-002 also had multiple confounding factors that led to inconsistent results, indicating a 9 out of 10 for LBP VAS at the 42-day visit, 7 out of 10 at the 90-day visit, and a 5 out of 10 for LBP VAS (a decrease in 3 scores from screening) at the 180-day visit. 

As the study was first-in-human, the researchers were limited to treating one diseased disc per participant by the ethics committee. Five out of the six participants had multiple diseased discs. The one participant who had a single diseased disc (participant 01-002) reported remarkable results. Participant 01-002 had been managing their pain using medication for 8.8 years. He was able to cease taking his pain medication from 90 days post-procedure onwards and maintained reduced pain levels throughout the end of the study. The participant scored a 5 out of 10 VAS for LBP and 34 for ODI at baseline. He subsequently reported 0 pain from day 42 to the end of the study at day 180 post-procedure. Additionally, he reported a 2 for ODI at the 42-day visit and 0 for the 90-day and 180-day visits. The participant's overall health score went from 45 at baseline to 95 at the study exit.

## Conclusions

The results from this pilot study show that DiscSeal^TM^ is safe and well-tolerated with early efficacy, indicating that DiscSeal^TM^ may be a promising minimally invasive treatment option for people suffering from discogenic LBP, who have tried and have failed first-line therapy. Due to the small sample size, the confidence in the results are limited, and therefore a larger, longer and statistically powered study that allows multiple diseased, pain-generating discs treatment, should be conducted.
